# Detection of dengue virus serotypes 1, 2 and 3 in selected regions of Kenya: 2011–2014

**DOI:** 10.1186/s12985-016-0641-0

**Published:** 2016-11-04

**Authors:** Limbaso Konongoi, Victor Ofula, Albert Nyunja, Samuel Owaka, Hellen Koka, Albina Makio, Edith Koskei, Fredrick Eyase, Daniel Langat, Randal J. Schoepp, Cynthia Ann Rossi, Ian Njeru, Rodney Coldren, Rosemary Sang

**Affiliations:** 1 Kenya Medical Research Institute, P. O Box 54628-00200, Nairobi, Kenya; 2 United States Army Medical Research Directorate, P. O Box 606, Nairobi, Kenya; 3 Kenya Ministry of Health -Division of Disease Surveillance and Response, P.O. Box 20781-00202, Nairobi, Kenya; 4 United States Army Medical Research Institute of Infectious Diseases (USAMRIID), 1425 Porter Street, Fort Detrick, Frederick, MD 21702-5011 USA

**Keywords:** Dengue, Serotypes 1, 2 and 3, Kenya

## Abstract

**Background:**

Dengue fever, a mosquito-borne disease, is associated with illness of varying severity in countries in the tropics and sub tropics. Dengue cases continue to be detected more frequently and its geographic range continues to expand. We report the largest documented laboratory confirmed circulation of dengue virus in parts of Kenya since 1982.

**Methods:**

From September 2011 to December 2014, 868 samples from febrile patients were received from hospitals in Nairobi, northern and coastal Kenya. The immunoglobulin M enzyme linked immunosorbent assay (IgM ELISA) was used to test for the presence of IgM antibodies against dengue, yellow fever, West Nile and Zika. Reverse transcription polymerase chain reaction (RT-PCR) utilizing flavivirus family, yellow fever, West Nile, consensus and sero type dengue primers were used to detect acute arbovirus infections and determine the infecting serotypes. Representative samples of PCR positive samples for each of the three dengue serotypes detected were sequenced to confirm circulation of the various dengue serotypes.

**Results:**

Forty percent (345/868) of the samples tested positive for dengue by either IgM ELISA (14.6 %) or by RT-PCR (25.1 %). Three dengue serotypes 1–3 (DENV1-3) were detected by serotype specific RT-PCR and sequencing with their numbers varying from year to year and by region. The overall predominant serotype detected from 2011–2014 was DENV1 accounting for 44 % (96/218) of all the serotypes detected, followed by DENV2 accounting for 38.5 % (84/218) and then DENV3 which accounted for 17.4 % (38/218). Yellow fever, West Nile and Zika was not detected in any of the samples tested.

**Conclusion:**

From 2011–2014 serotypes 1, 2 and 3 were detected in the Northern and Coastal parts of Kenya. This confirmed the occurrence of cases and active circulation of dengue in parts of Kenya. These results have documented three circulating serotypes and highlight the need for the establishment of active dengue surveillance to continuously detect cases, circulating serotypes, and determine dengue fever disease burden in the country and region.

## Background

Dengue fever is regarded as the most important re-emerging mosquito-borne disease globally and is endemic in more than 125 countries worldwide [1]. It is an acute systemic viral illness that manifests with varying degrees of severity ranging from a mild febrile illness to severe hemorrhagic presentations, dengue hemorrhagic fever (DHF) or dengue shock syndrome (DSS). Dengue viruses are mosquito-borne members of the *Flavivirus* genus, family *Flaviviridae*, first isolated in 1943 and 1945 in Japan and Hawaii respectively [2]. Dengue fever is caused by infection with one of four distinct dengue serotypes (DENV1 to DENV4) that are genetically related but antigenically distinct and with extensive genetic diversity within the different serotypes. Immunity is serotype specific and there is no cross protection between the serotypes [3]. It is estimated that 96 million apparent dengue infections occurred worldwide in 2010 with most of these being reported in Asia, which bore 70 % of the global burden while Africa bore 16 % of the global burden. There are more than 390 million cases annually, of which 294 million maybe in apparent infections not detected by the public health system [4].

In existence for centuries, the Chinese documented symptoms compatible with dengue in 992 AD and associated the disease with flying insects and water [5]. It was not until the 20^th^ century when the viral etiology and the role of mosquitoes in its transmission were determined [1]. *Aedes aegypti* (*A. aegypti*), the main arthropod vector for dengue has its origins in Africa and is wide spread in Africa and the tropics. The mosquito has a high affinity for human blood, a high adaptation to urban dwelling in close proximity to human settlements, and a high vectorial capacity for the four dengue serotypes [6]. Rapid urbanization and globalization is associated with the expansion of dengue fever in the 20^th^ century [7]. It breeds in and around houses in regular water containers or disposed water-holding vessels. Due to its limited flight range the female *A. aegypti* persists in a domesticated environment contributing to the spread of dengue through high human-mosquito-human contact within communities [8].

The first documented dengue outbreak in Africa occurred in Durban, South Africa in 1927 as determined by a retrospective serological study [9]. Subsequently, dengue virus isolations in Africa have been reported in 1964–68 in Nigeria (DENV1 and 2) [10], in 1983–85 in Mozambique (DENV3) [11], in 1984 in Sudan (DENV1 and 2) [12] and in 1986 in Senegal (DENV4) [13]. In the past five decades sporadic or epidemic cases of dengue have been increasing in sub-Saharan Africa with 22 countries reporting outbreaks. East Africa has experienced the largest burden in this period with outbreaks occurring in the Island nations of Réunion (1977–1978), the Seychelles (1977–1979), the Comoros (1992–1993), and Cape Verde (2009). In addition Djibouti also recorded a large outbreak in 1992–1993. Approximately 300,000 cases were detected in these 5 outbreaks. Dengue is currently endemic in 34 African countries with transmission being reported through local disease transmission, detection of laboratory confirmed cases, and detection among travelers returning to countries not endemic to dengue [14].

In Kenya, the first documented dengue outbreak (DENV2) occurred in 1982 in the coastal cities of Malindi and Mombasa and was thought to have spread from an outbreak that had occurred in the Seychelles in 1979–1980 [15]. Subsequently, although dengue outbreaks were documented in the neighboring countries of Somalia, Djibouti and South Sudan [14, 16], only rare sporadic cases of DENV2 were detected in the coastal town of Mombasa. Seroprevalence studies performed in Kenya have indicated high prevalence of dengue in coastal Malindi at 34.17 % and lower prevalence in western Busia at 1.96 % [17]. Due to lack of active surveillance and reporting structures for dengue infections in much of East Africa, there is a lack of appreciation of the burden of the disease in the region and detection of cases is often hampered by non-specific clinical manifestation of the illness, which mimics other common fever causing illnesses like malaria and typhoid fever and the unavailability of diagnostic capabilities in most of the health centers.

In the continued absence of a viable/approved vaccine, the prevention and control of dengue is currently reliant on vector control methods and early detection of cases through continued surveillance that trigger mosquito control activities to alleviate human suffering and emergence of severe disease caused by widespread virus transmission of multiple serotypes.

In September 2011, reports of increased cases of acute febrile illness were reported in Mandera in northeastern Kenya bordering Somalia. In the subsequent months and years, the viral hemorrhagic fever (VHF) laboratory at the Kenya Medical Research Institute (KEMRI) continued to receive samples from northeastern Kenya and from Mombasa on the Kenya coast for dengue fever testing.

**Fig. 1:**
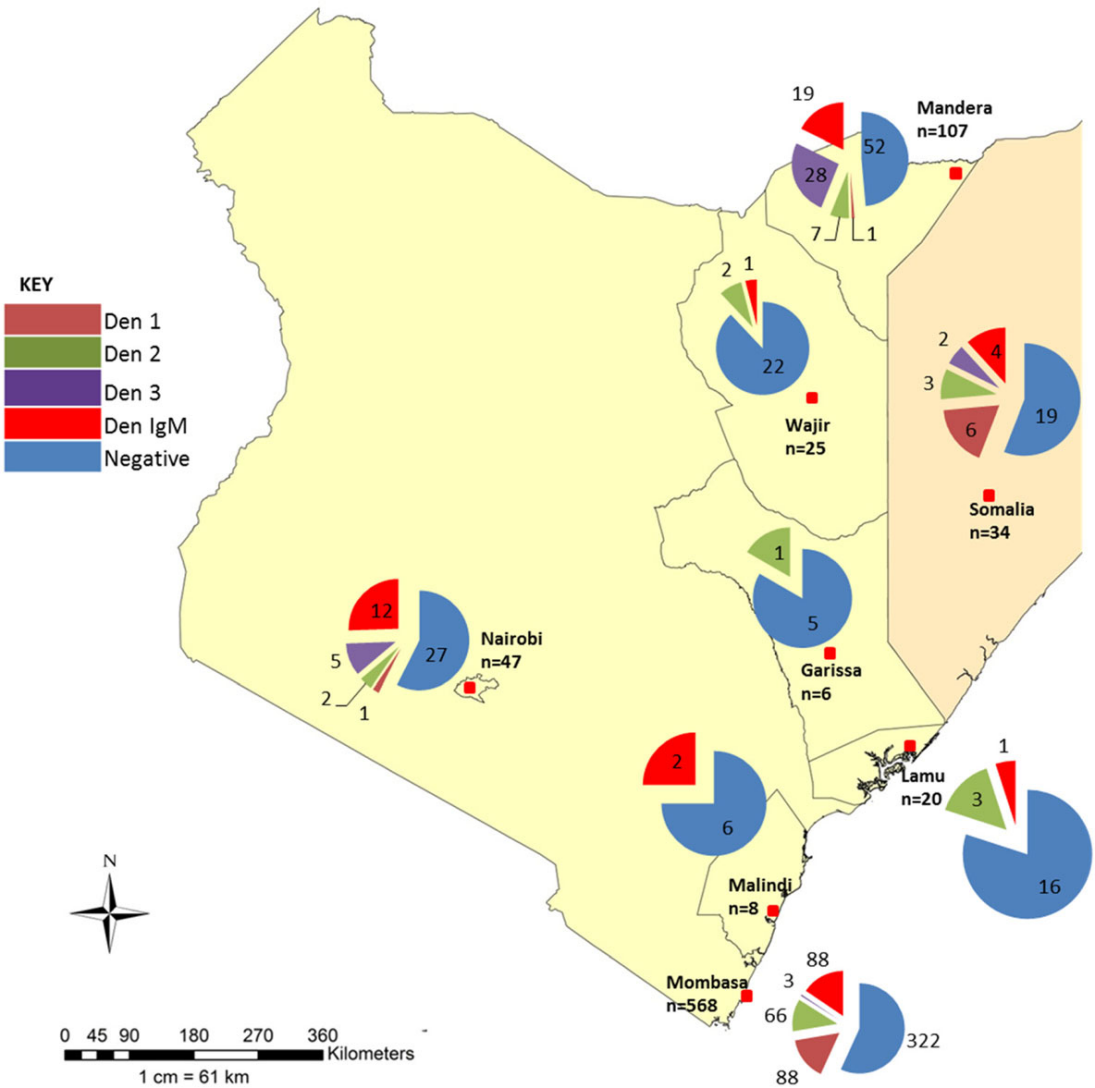
Map of Kenya showing locations from which dengue cases were detected, 2011–2014. Map of Kenya showing the regions and number of samples received from different parts of Kenya. Samples were received from 7 regions of Kenya and from neighboring Somalia. Dengue serotypes 1–3 represented in different colours in the map were detected in various regions as shown in the pie charts. Only sites from which dengue cases were detected are shown on this map

Laboratory testing was conducted with the support of the Global Emerging Infections Surveillance (GEIS) program of the United States Army Medical Research Directorate Kenya (USAMRD-K). The laboratory responds to reports of suspected arbovirus/VHF infections in Kenya and on request of the World Health Organization (WHO) other countries neighboring Kenya that lack laboratory capacity by performing diagnostic testing on samples of suspected cases of arboviruses and VHF infections. From September 2011 to December 2014, as part of the Kenya Ministry of Health response effort, the laboratory received samples from diverse private and government health facilities in northeastern Kenya in Mandera and Wajir counties, hospitals in the capital city of Nairobi and from both private and government hospitals in Mombasa, Malindi and Lamu along the Kenyan coast (Fig. 1).

## Methods

### Study population and sample collection

Samples were collected from patients of both sexes and all ages who presented with a sudden onset of fever accompanied by body aches. Following the detection of initial dengue cases a clinical working case definition was developed by the Division of Disease Surveillance and Response, Ministry of Health and sent out to all health facilities in the affected and high risk areas.

### Sample collection and testing

Venous blood was collected in vacutainer tubes with no anticoagulant using standard phlebotomy practices from patients that met the case definition. Samples were transported to the laboratory in cold storage where they were centrifuged and serum obtained for testing. All samples were tested using the IgM antibody capture enzyme-linked immunosorbent assay (MAC-ELISA) to detect IgM antibodies against dengue, yellow Fever and West Nile. A subset of the samples was tested for exposure to Zika using a commercial IgM kit. Flavivirus family, yellow fever, West Nile, dengue consensus and dengue serotype specific RT-PCR primers were used to detect an acute infection and to determine the infecting serotype. Representative samples of RT-PCR positive samples for each of the three dengue serotypes detected were sequenced to confirm circulation of the various dengue serotypes.

### Laboratory analysis

#### MAC-enzyme linked immunosorbent assay

The IgM antibody capture ELISA (MAC-ELISA) used to detect presence of IgM antibodies was a laboratory derived test (LDT) provided by the Diagnostic Systems Division, United States Army Medical Research Institute of Infectious Diseases (USAMRIID). A 96 well Immunolon plate (Nunc, Denmark) was coated with a commercial anti-human IgM antibody that reacts specifically with human IgM, (goat anti-human IgM, Kirkegaard and Perry laboratories Gaithersburg, MD, USA) and incubated at +4 °C for 12–16 h. The plate was washed using a wash buffer (PBS, pH 7.4, 0.01 Merthiolate, 0.1 Tween-20). This was followed by addition of the dengue IgM positive control, negative control and sample all diluted 1:100 in diluent buffer (PBS, pH 7.4, 0.01 Merthiolate, 0.1 Tween-20, 5 % skim milk). Plates were incubated at 37 °C for one hour. The plate was then washed and 100 μl of dengue antigen solution consisting of equal amounts of inactivated lyophilized dengue fever virus 1–4 added in one half of the test wells and a corresponding negative antigen (same dilution) is added in the other half of the test wells.

Dengue antigens used in the assay were obtained from various sources. Dengue 1 - Hawaii isolated in 1944 from a human [18], dengue 2 - New Guinea C, isolated in 1944 from a human [19] dengue 3 - H87, Philippines isolated in 1956 from a human and dengue 4 - H241, Philippines isolated in 1956 from a human [20]. The IgM antigens were supernatants from vero cells infected with the appropriate isolate and supernatants were inactivated using 0.3 % beta-propiolactone and cobalt irradiated using 3 million rads and safety tested. The plate was incubated for one hour at 37 °C. After washing 100 μl dengue specific detector antibodies (anti-dengue hyperimmune mouse ascitic fluid) was added to each well and incubated for one hour at 37 °C. The plate was washed and 100 μl of HRP labelled goat anti-mouse IgG, heavy and light chain specific conjugate that reacts specifically with mouse IgG (Kirkergard and Perry, catalog 074–1806) added in all the wells and plate incubated for one hour at 37 °C. The plate was then washed and 100 μl of ABTS substrate (Kirkergard and Perry, Cat. No. N8 50-62-00, Gaithersburg, MD) was added and the plate incubated at 37 °C for 30 min. The reaction was visualized by a green colour and the optical density (OD value) was read with a spectrophotometer at 405 nm. The adjusted OD was calculated by subtracting the OD of the negative/mock antigen coated wells from the positive antigen coated wells. The OD cut-off was calculated as the mean of the adjusted OD of the negative control sera plus three times the standard deviations. All samples were also tested for IgM antibodies against West Nile and yellow fever using the same procedure as outlined for dengue above but with variations in the positive controls, the positive and mock antigens and the virus specific detector antibodies. The yellow fever antigen used was obtained from the Asibi strain isolated from a human in Ghana in 1927 [21] while the West Nile antigen was the Eg101 strain isolated in Egypt in 1951 [22].

To rule out cross reactivity with Zika, 15 randomly picked samples that tested positive for dengue IgM antibodies were screened using the Euroimmun Anti-Zika virus IgM ELISA (Euroimmun, Lübeck, Germany) kit following the manufacturer’s instructions. Briefly the serum was diluted 1:101 in sample buffer, incubated at room temperature for 10 min, added into the appropriate microplate wells and incubated at 37 °C for 1 h. This was followed by addition of a peroxidase labelled anti human IgM conjugate, substrate, and finally a stop solution while performing the wash steps in between incubations and adhering to the appropriate incubation temperatures for each step. The optical density (OD) was measured using an ELx800™ absorbance microplate reader (Biotek Winooski, Vermont, USA). A cut-off ratio was calculated, and values <0.8 were regarded as negative, ≥0.8 to <1.1 as borderline, and ≥ 1.1 as positive [23].

#### Nucleic acid (RNA) extraction

Viral RNA was extracted using the QIAamp Viral RNA Minikit (QIAGEN, Hilden Germany) according to the manufacture’s protocol. A final volume of 60 μL of RNA was obtained and used as a template for cDNA synthesis and for the subsequent PCR reactions.

#### cDNA synthesis from viral RNA

To convert extracted RNA to cDNA, 10 μL of the extracted sample viral RNA was mixed with 2 μL of 50 ng/μL random hexamer primer in a 0.2 ml PCR tube. The mixture was incubated in a thermocycler for 10 min at 70 °C. The reaction was stopped and the following components added to the PCR tube: 4 μL of 5X First Strand Buffer (Invitrogen), 1 μl of 10 mM dNTPs, 2 μl of 100 mM DTT, 0.25 μl of RNAse Inhibitor (40U/μl) and 1 μl of Superscript III Reverse transcriptase (200 U/μl). The mixture was then placed in a thermocycler set at the following conditions: 25 °C for 15 min, 50 °C for 50 min, followed by 70 °C for 15 min in the thermocycler and 4 °C hold temperature. A total of 20 μL of cDNA was obtained.

The PCR amplification of targeted viral sequences in the cDNA was performed in a 25-μL reaction containing: 12.5 μl of Amplitaq Gold 360 PCR master mix (Applied Biosystems USA), 50 picomoles each of forward and reverse primer, 2 μl of the cDNA and 9.5 μl of DEPC treated water to top up to 25 μl. Samples were first tested using flavivirus family primers. Samples testing positive with flavivirus family primers were further tested with yellow fever, West Nile and consensus dengue primers D1 and D2. Samples testing positive with the dengue consensus primers that target the E/NS1 junction of the virus genome were further tested for the 4 dengue sero types using the appropriate primers (Table 1).

**Table 1:** Primer sequences used for flavivirus, yellow fever, West Nile, dengue consensus and sero type specific RT-PCR reactions

Primer	Sequence	Base pair size of amplified product	Reference
FU1 CFD3	5′- TAC AAC ATG ATG GGA AAG AGA GAG AA-3′ 5′- GTG TCC CAG CCG GCG GTG TCA TCA GC-3′	260 Flavivirus	[46]
KP7 KP81	5′-GCA GAG TGA TCG ACA GCC G-3′ 5′-CCA CCA GAC CAT TCG GCA TG-3′	258 West Nile	[47]
YF7 R CAGF	5′- AAT GCT CCC TTT CCC AAA TA- 3′ 5′- CGA GTT GCT AGG CAA TAA ACA CAT TTG GA-3	670 Yellow Fever	[48]
D1 D2	5′-TCAATATGCTGAAACGCGCGAGAAACCG-3′ 5′-TTGCACCAACAGTCAATGTCTTCAGGTTC-3′	511 Dengue consensus	[49]
TS1	5′-CGTCTCAGTGATCCGGGGG-3′	482 (Dl and TS1) DENV1	[49]
TS2	5′-CGCCACAAGGGCCATGAACAG-3′	119 (Dl and TS2) DENV2	[49]
TS3	5′-TAACATCATCATGAGACAGAGC-3′	290 (Dl and TS3) DENV3	[49]
TS4	5′-CTCTGTTGTCTTAAACAAGAGA-3′	392 (Dl and TS4) DENV4	[49]

The primer sequences above were used to detect exposure to the various arboviruses using amplification conditions as described in the corresponding references for each primer listed.

A positive control cDNA and a negative control were included during the setting up of all PCR reactions. Electrophoresis of the amplified DNA products was done on a 1–2 % agarose gel in 1 % Tris-borate EDTA buffer stained with ethidium bromide. The PCR product bands were visualized by a UV trans illuminator and recorded using a gel photo imaging system.

#### Sequencing and phylogenetic analysis

Amplified target DNA bands were either purified directly from the PCR reaction or from the gel using Wizard® SV Gel and PCR Clean-Up System kit (Promega Madison, WI, USA). Sequencing was outsourced and performed using ABI-PRISM 3130 Genetic Analyzer (Applied Biosystems, Foster City, CA). Both forward and reverse strands were sequenced and the raw chromatogram file was edited for bad calls using DNAbaser v.3.0.The sequences were compared with available sequences using Basic Local Alignment Search Tool and the GenBank database to confirm the identity of the virus isolate. The sequences were aligned using Muscle [24] in Molecular Evolutionary Genetics Analysis (MEGA) software version 76 was used for phylogenetic analysis using the Maximum likelihood statistical method tested with 1000 bootstrap replicates based on the Tamura-Nei model [25]. The phylogenetic tree was inferred in MEGA version 7. A total of 15 (5 for each serotype DENV1-3) samples were sequenced.

## Results

From July 2011 to December 2014 a total of 868 clinical samples obtained from febrile cases were tested. The least number of samples were received in 2012 (32/868) while the year 2013 accounted for the highest number of samples received (567/868).

Out of the 868, 40 % (345/868) of the samples were positive for dengue; 14.6 % (127/868) were positive for dengue IgM antibodies while 25.1 % (218/868) were positive for various dengue serotypes by RT-PCR (Table 2).

**Table 2:** Number of dengue positives cases detected in Kenya; 2011-2014

	Numbers detected				
Year	DENV1	DENV2	DENV3	Den IgM	Negative
2011	6	3	32	19	69
2012	0	9	2	6	15
2013	89	60	4	70	344
2014	1	12	0	32	95
Total	96	84	38	127	523

A total of 868 samples were tested in the 4 year period and numbers of dengue positives and serotypes detected varied in the different years.

Of the 345 samples that tested positive for dengue 6 % (21/345) tested positive for dengue by both IgM ELISA and RT-PCR.

Overall, 68 % (588/868) of samples were from male patients and 32 % (280/868) were from female patients). The 21–50 year age group accounted for 52 % (454/868) of all the samples received and 49.8 % (172/345) of all dengue positive cases detected.

**Fig. 2:**
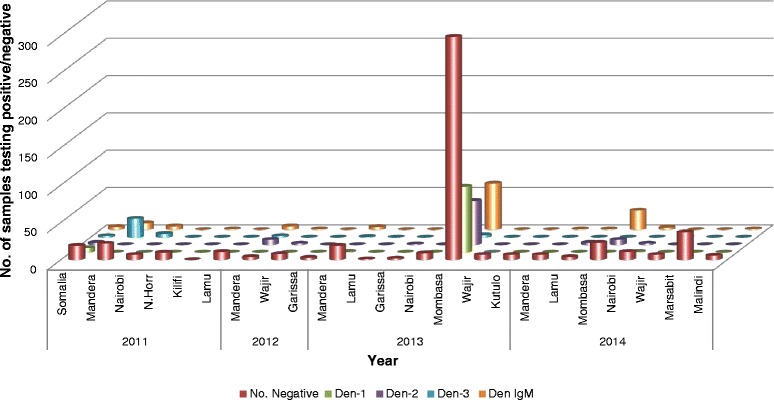
Number of dengue cases per region in Kenya, 2011–2014. Distribution of DENV serotypes in various regions in Kenya varied from 2011 to 2014. Overall, DENV1 was the most dominant serotype followed by DENV2 and 3 respectively. The colour green, purple, blue and orange represent the number of DENV-1, DENV-2, DENV-3 and IgM cases detected respectively

Three dengue serotypes were detected during this period (DENV1-3) with no case of DENV4 being detected. Serotypes detected varied by year and region with DENV1 accounting for 44 % (96/218) of the three serotypes detected followed by DENV2 at 38.5 % (84/218) and DENV3 17.% (38/218) detected in both the northern and coastal regions of Kenya (Fig. 2).

Samples from the coast predominantly tested positive for DENV1 (88/160) followed by DENV 2 (69/160) with the two serotypes accounting for 98 % of all the serotypes from the coast. In northeastern Kenya, DENV 3 was dominant accounting for 72 % (28/39) of all the DENV serotypes detected during this reporting period in the region (Fig. 2).

In 2011, 129 samples were received from six facilities with most of the samples coming between September and November from northeastern region and Nairobi accounting for 15 % (129/868) of all the samples tested 2011 to 2014. Overall, 46.5 % (60/129) of samples received were positive for dengue; 32 % (41/129) were positive by RT-PCR and 15 % (19/129) were positive by MAC ELISA. DENV3, DENV1 and DENV2 accounted for 78 % (32/41), 15 % (6/41) and 7 % (3/41) of all the PCR positive samples, respectively. A subset of 34 samples was received from Somalia in July 2011 of which 44 % (15/34) tested positive for DENV1-3 and IgM antibodies (Fig. 2).

In 2012 there was a drop in the number of samples received. Only 32 samples were received from three facilities mostly between February and April from northeastern region. It is not clear what factors were associated with the sudden reduced numbers of samples received at the laboratory. We speculate that it may not have been associated with a sudden drop in patients with fever but more due to the public health response measures following the detection of cases in 2011. Detection of cases in 2011 was followed by dispatch of response teams tasked with initiating community sensitization on infection prevention, mosquito control activities and supplied local health clinics with rapid dengue diagnostic kits hence samples were tested at the respective sites. The samples accounted for 4 % (32/868) of the total samples received and 53 % (17/32) tested positive for dengue; 34 % (11/32) were positive by RT-PCR with DENV2 accounting for 82 % (9/11) and DENV3 accounting for 18 % (2/11) of the PCR positives (Fig. 2).

In 2013, a total of 567 samples were received from seven facilities, with most coming between April and May accounting for 65 % of all the samples received. The majority of samples came from Mombasa accounting for 89 % (507/567) of all samples received that year and 57 % (507/864) of all the samples received over the four year study. The remaining samples came from northeastern Kenya. Overall, 39.3 % (223/567) of the samples were positive for dengue by either RT-PCR or IgM ELISA. DENV1 and DENV2 accounted for 39.9 % (89/223) and 26.9 % (60/223) of the positive cases, respectively. DENV3 was detected in 1.7 % (4/223) of the positive cases (Fig. 2).

In 2014, a total of 140 samples from seven facilities were received mostly from the coast of which 32 % (45/140) tested positive for dengue; 71 % (32/45) were detected by IgM ELISA and DENV2 and DENV1 accounted for 26.6 % (12/45) and 2.2 % (1/45) of the PCR positive samples, respectively (Fig. 2).

All positive patient samples collected from Nairobi had a travel history to either the northern, eastern, or coastal parts of Kenya where active transmission was ongoing during the surveillance period. DENV1-3 was detected in Nairobi during this period, but there was no evidence of active transmission documented.

Co-infection with more than one serotype was detected in two samples. Co-infection with DENV2 and 3 was detected in one sample collected in Mandera in the early stages of the outbreak in 2011 and the second in a sample from Mombasa that also had both DENV2 and 3 in 2013. Co infections with other flaviviruses (yellow fever and West Nile) were not detected by PCR in any of the 868 samples tested. The IgM assays performed did not detect yellow fever, West Nile and Zika in the samples tested.

**Fig. 3:**
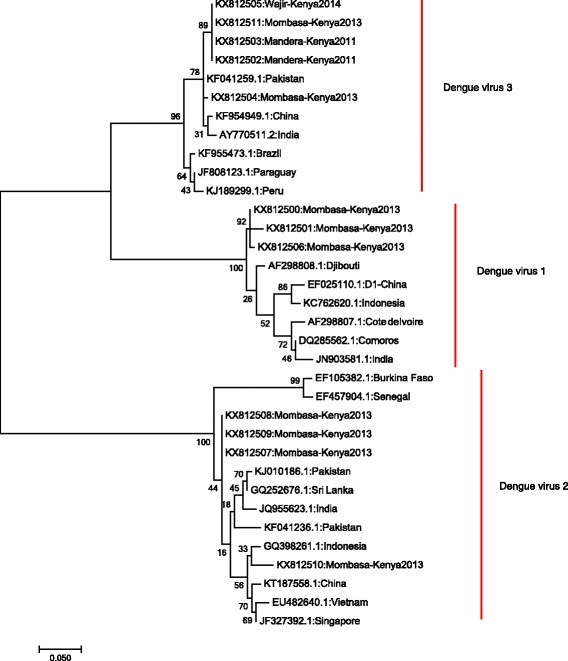
Maximum Likelihood tree of Dengue virus capsid sequences. Phylogenetic relationships of Kenyan isolates of DENV-1, DENV-2 and DENV-3 as inferred by using the Maximum Likelihood method based on the Tamura-Nei model. The percentage of trees in which the associated taxa clustered together is shown next to the branches. Bootstrap values above 80 % are highlighted. The tree is drawn to scale, with branch lengths measured in the number of substitutions per site. The analysis involved 34 nucleotide sequences. There were a total of 200 positions in the final dataset. Evolutionary analyses were conducted in MEGA7

Of the 15 samples sequenced, 12 samples gave good quality reads and 5 were able to sequence the full 511 base pair region containing capsid and pre M genes. The remaining seven had good quality sequences for the capsid gene. The 12 sequences were trimmed to remain with capsid gene which was used for phylogenetic analysis. Phylogenetic analysis of the capsid gene sequences for representative selected PCR positive samples from the cases revealed that DENV1 isolates from Mombasa (2013) showed close relatedness to a DENV1 isolate from Djibouti isolated in 1998. All the DENV2 isolates from Kenya detected in Mombasa in 2013 showed close relatedness to isolates detected in different parts of Asia. All DENV3 isolates, two from Mandera (2011), two from Mombasa (2013) and one from Wajir (2014) were closely related to a DENV3 isolates from Pakistan, China and India obtained in the year 2006, 2013 and 2009, respectively (Fig. 3).

## Discussion

Although considered endemic in Africa and Kenya [14], there has been limited information documenting active dengue virus transmission in Kenya among the human population since the early 1980s. Current available information has relied on serological surveys [17] and has not described the circulating serotypes in the country. With 50 % of the world’s population living in dengue endemic countries, Africa included; the continent continues to face challenges in case detection and reporting resulting in limited information available towards understanding the true disease burden and economic impact of dengue. Lack of awareness of dengue among health workers, erratic treatment seeking behavior among populations, presence of symptomatically similar illnesses like malaria and typhoid, low case fatality rates, limited availability appropriate diagnostic systems, and under reporting by existing public health systems all contribute to under recognition of dengue in the continent [1, 26].

Results during this 4 year period were able to detect multiple dengue serotypes and helped to provide clear evidence of active dengue transmission and identified serotypes circulating in parts of northeastern and coastal Kenya.

All four dengue serotypes have been detected in Africa [14]. Our laboratory based results showed that in parts of Kenya DENV1 and 2 were most dominant. This is consistent with literature suggesting most epidemics in Africa are caused by serotypes 1 and 2 [14, 16]. Infection with DENV4 is less common, but it has been documented in parts of Africa [13] and in Europe from travelers returning from Africa [27]. DENV4 was not detected in any of the 868 cases tested in this period. It is unclear why DENV4 was not in circulation. Since the serotype is associated with mild clinical disease, the absence of complications may have resulted in patients not seeking treatment; hence it would often go undetected where it occurs [28].

Detection of the first dengue cases in northeastern Kenya in September 2011 was preceded by dengue detection in samples from Mogadishu, Somalia in February 2011, which suggested there was active transmission of dengue going on in Somalia. From the Somali samples, three serotypes (DENV1-3) were detected. In the Kenya 2011 cases, only DENV3 was detected in samples from Mandera in northeastern Kenya. By 2013, DENV1-3 was being detected in samples from the northern part of Kenya. It is reasonable that the cases in Mandera on the border with Somalia may have resulted from infected travelers from Somalia.

Kenya and Somalia share a long porous border where communities freely interact in search of pasture and other economic activities. Though it may be assumed that the infection spread from Somalia into Kenya, it is not clear why there was a six month gap between the Somali cases and the first detection in northeastern Kenya. It may be that the first cases went undetected or were misdiagnosed as malaria. Considering that no severe dengue infections were detected in Kenya and that the infections are self-limiting, the initial cases may have resolved only to be detected much later when it affected large populations concentrated in the major town of Mandera. All the three serotypes detected in Somalia were also detected in northeastern Kenya. Somalia is currently hosting peace keeping forces from various parts of the world. The forces present a naïve population and several outbreaks among peace keeping forces have been documented [29]. Cross border dengue infections is of concern among many countries since it is considered a major source of dengue spread [30].

Urbanization and infrastructure connectivity has been shown to be a major factor facilitating the spread of dengue infections between affected and non-affected areas [6]. Mandera is home to the Kenya Somali ethnic community who practice pastoral farming, but live in urban setting in Mandera town. The town lacks piped water, relying on water collected from a nearby river or occasional rainfall. The water is stored in large concrete water cisterns and other artificial containers that are perfect breeding sites for *Aedes aegypti*, the primary vector of dengue viruses.

Since the first detection of dengue in 1982, coastal Kenya has long been suspected of being a dengue endemic zone. Numerous studies have attempted to show dengue circulation in human and vector populations and the presence of competent vectors [17, 31]. This study documented the circulation of multiple dengue serotypes (DENV1-3) in Kenya and has confirmed the presence of ongoing virus transmission.

Dengue fever cases were identified in Nairobi, the capital city of Kenya, however all cases had a prior history of recent travel to the dengue affected areas of northeastern and coastal Kenya. Despite detection of acute cases in Nairobi, there was no evidence of active dengue transmission. It is not clear why this was so, but we speculate that the numbers of acute cases (eight) could have been too few and spread apart for the establishment of active local transmission. In addition, possible inherent differences in the vector competence capabilities to dengue virus of the Nairobi *Ae. aegypti* mosquito population compared to populations in other regions with active cases coupled with other environmental factors may have played a role in the lack of establishment of potential active transmission [31].

No case of DHF/DSS was detected during the surveillance period despite the co-circulation of multiple serotypes (DENV1-3), a phenomenon commonly associated with DHF/DSS. The primary infecting serotype determines severity of the infection. Primary infections with DENV1 and 3 tend to cause more severe clinical disease manifestations, while DENV2 and 4 are associated with increased severity when they occur as secondary infections [32] Co-infection with more than one serotype was detected in two samples. Co-infection with DENV2 and 3 was detected in a sample collected in Mandera in the early stages of the outbreak in 2011 and a sample from Mombasa with co-infection of DENV2 and 3 was detected in 2013. Due to logistical constraints, the laboratory was not able to ascertain the outcome on these patients. Previous studies have also shown that race may also play a role in offering partial protection against severe forms of dengue. Genetic polymorphisms that offer partial protection against severe forms of dengue have been identified in people of African descent [33, 34]. This could have played a role, but cannot be substantiated from laboratory based surveillance.

Clinical outcomes of dengue cases may be influenced by the circulation of multiple DENV serotypes and is considered a factor in the reemergence of dengue hemorrhagic fever [35]. Co-circulation of various DENV serotypes is well documented as a frequent occurrence in various parts of the world. The outbreak in Kenya was characterized by the detection of multiple serotypes with the predominant serotype being DENV1, followed by DENV2 and then DENV3, which is similar to most dengue outbreaks detected globally where multiple serotypes are detected [36, 37].

The detection of multiple dengue serotypes in Kenya with close relatedness to isolates obtained in other parts of Africa, South and South East Asia shows the continued movement and the wide geographic range of the dengue serotypes. Only DENV1 isolates showed any close relatedness to an African isolate from Djibouti (AF298808), which has been shown to be more genetically related to Asian isolates than to African isolates [38]. All DENV2 and 3 isolates showed relatedness to Asian isolates indicating transmission and sustenance in countries away from the initial geographic origin.

Over the last decade, Kenya has developed into a major air and sea port transport hub in the region connecting the Asian and African continents for commercial and tourist purposes. Increased travel between affected and non-affected areas constitutes a constant threat with travelers acting as vehicles of disease spread. In addition rapid urbanization and globalization is associated with the expansion of dengue transmission by providing a conducive environment for the mosquito vector [39, 40].

Of concern were the 60 % of the samples that tested negative for dengue, West Nile and yellow fever viruses by IgM ELISA and RT-PCR despite being collected from patients presenting with fever. This demonstrates the need to constantly review and avail comprehensive differential disease diagnostic panels at health facilities where possible and at the testing laboratories. This will enhance detection of underlying or co circulating reemerging and emerging disease threats caused by parasitic, viral, bacterial or other pathogens associated with febrile illness manifestations in human populations. An opportunity to determine the etiology of arbovirus infections is often missed as fevers caused by arboviruses may be misdiagnosed as malaria or Vis versa. In addition, overlaps in geographical locations and concurrent infections of arboviruses from the same or different families are well documented in various parts of the world and Africa [40–45]. This highlights the need for continued vigilance and review of the existing testing algorithms for diseases associated with febrile manifestations.

In this reporting period, we were only able to screen a small subset of samples (12 % of the dengue IgM positives) for cross reactivity with Zika for logistical reasons. Although all the samples tested negative for Zika IgM antibodies, our results may be biased towards dengue as we were not able to screen for Zika in all the samples that tested positive for dengue IgM antibodies.

As dengue becomes endemic in Kenya, health care providers are increasingly aware of the need to quickly detect infection and provide appropriate care to patients. The availability of rapid diagnostic kits at health facilities has resulted in the reduced flow of samples to the KEMRI laboratory, but cases continue to be detected in the northern and coastal regions of Kenya.

## Conclusion

Confirmatory laboratory diagnosis in Kenya facilitated the detection of dengue virus circulation in the northern and coastal regions of Kenya and in the capital city Nairobi. Early laboratory detection allows clinicians to institute supportive treatment for better prognosis.

There is need to establish on-going dengue surveillance to continuously detect outbreaks, the serotypes circulating, and determine dengue fever disease burden in the region. Seasonal variation should also be established to identify high risk times and facilitate appropriate public health responses. Circulation of multiple serotypes may also lead to increased cases of severe form of dengue.
